# Neuroimaging and clinicopathological differences between tumefactive demyelinating lesions and sentinel lesions of primary central nervous system lymphoma

**DOI:** 10.3389/fimmu.2022.986473

**Published:** 2022-08-18

**Authors:** Chenjing Sun, Jinming Han, Ye Lin, Xiaokun Qi, Changqing Li, Jianguo Liu, Feng Qiu

**Affiliations:** ^1^ Senior Department of Neurology, The First Medical Center of PLA General Hospital, Beijing, China; ^2^ Department of Neurology, Xuanwu Hospital, Capital Medical University, Beijing, China; ^3^ Department of Neurology, Beijing Chaoyang Hospital, Capital Medical University, Beijing, China

**Keywords:** tumefactive demyelinating lesions, primary central nervous system lymphoma, sentinel lesions, pathology, neuroimaging, diagnosis

## Abstract

**Objective:**

It is still a challenge to distinguish sentinel lesions of primary central nervous system lymphoma (PCNSL) from atypical tumefactive demyelinating lesions (TDLs) in clinical practice. We aimed to investigate potential differences of clinical features, neuroimaging findings and pathological characteristics between PCNSL and TDLs, improving early accurate diagnosis.

**Methods:**

It was a retrospective study involving 116 patients with TDLs and 150 patients with PCNSLs. All cases were pathologically confirmed. Clinical features, neuroimaging findings and pathological characteristics between two groups were analyzed.

**Results:**

The onset age was 37 ± 14 years in TDLs and 58 ± 13 years in PCNSL(p=0.000). Main onset symptom was headache in TDLs, while cognitive impairment was frequently noted in PCNSL. CT brain scan image showed hypodense lesions in most cases of TDL (110/116, 94.8%), while approximately 80% patients (120/150) with PCNSL had hyperdense lesions. Furthermore, we found that the presence of Creutzfeldt-Peters cells (might be misdiagnosed as tumor cells) may serve as an important feature in TDLs.

**Conclusions:**

Onset age of patients with TDLs was younger than PCNSL. Neuroimaging features on brain CT scan might provide clues to make a differential diagnosis. Pathological features of PCNSL with sentinel lesions or following steroids therapy might mimic TDLs. Dynamic neuroimaging pathological and follow-up information were essential for an accurate diagnosis.

## Introduction

Tumefactive demyelinating lesions (TDLs), also called demyelinating pseudotumor (DPT), was first described by van der Velden and colleagues (1). TDLs are special inflammatory demyelinating lesions (> 20 mm in diameter) in the central nervous system (CNS), causing a variety of clinical manifestations. During an early disease stage, some patients only presented with cognitive impairment and TDLs (with surrounding edema or/and enhanced lesions) can be detected incidentally on magnetic resonance imaging (MRI) even. So TDLs are easily made as a suspected diagnosis of tumor (such as primary central nervous system lymphoma (PCNSL) or high-grade glioma) (2–4). PCNSL is a rare disease condition representing 6% of intracranial neoplasms and 1-2% of systemic lymphoma, with the plaques usually being involved the midline structure and white matter areas (5),. Main therapeutic approaches for PCNSL and TDLs are different. Shrinking PCNSL lesions can be noted following corticosteroids and TDLs can be easily misdiagnosed as PCNSL causing unreasonable treatment. Some PCNSL lesions can be appeared as demyelinating lesions during an early disease stage, described as a ‘sentinel lesion’ (6–8). Brain lesions following corticosteroids in PCNSL and sentinel lesions share similar pathological features and the final diagnosis is still challenging even after multiple times of biopsies. Previous studies regarding sentinel lesions of PCNSL and atypical TDLs were limited in reviews, case reports or small sample sizes (9–12). Therefore, investigating potential differences of clinical features, neuroimaging findings and pathological characteristics between PCNSL and TDLs is of importance.

## Methods

### Ethics statement

This study was conducted at the Sixth Medical Center of PLA General Hospital, Beijing, China. The patients/participants provided their written informed consent to participate in this study. This study was reviewed and approved by The Six Medical Center of PLA General Hospital, China.

### Patient information

A total of 116 patients with TDLs and 150 patients with PCNSL who were treated in the Sixth Medical Center of PLA General Hospital between January 1^st^, 2010 and January 1^st^ 2022 were included in this study. Detailed history including sex, onset age, onset neurological symptoms and treatment regimens were retrospectively reviewed and analyzed.

### Neuroimaging information

All patients were examined with a GE lightspeed 16-slince CT and GE Signa 1.5 or 3. 0 T MRI (General Electric, Milwaukee, WI, USA). The CT scan ranged from the canthomeatal line to calvarium. Brain window (window level: 35Hu, window width: 80hu) and bone window (window level: 450Hu, window width: 1500Hu). Scan parameters included tube voltage 130Kv, tube current:270mAs, 4.8mm slice thickness, andcontinuous scan 16 layers. The matrix was 512 * 512. Imaging slice thickness and interslice gap were same in all participants. Conventional imaging techniques were used: turbo spin-echo sequences for T_2 -_Weighted imaging (T_2_WI) (TR 4,660 ms; TE 110 ms; 6-mm slice thickness with a 2-mm interslice gap), FLAIR imaging (TR 9,000 ms; TE 120 ms; TI 2,200 ms, matrix 256 × 192, field of view (FOV) 240 mm; 6-mm slice thickness with a 2-mm interslice gap), T_1_-weighted (T_1_WI) T1-weighted imaging (TR 2415 ms; TE 13.9 ms; TI 750 ms; 6-mm slice thickness with a 2-mm interslice gap) and diffusion- weighted imaging (DWI) (TR 7000ms; TE 83.7 ms, b = 1,000 sec/mm^2^, 6-mm slice thickness with a 2-mm interslice gap). Contrast T_1_WI (TR 400-600 ms; TE 6-10 ms; matrix 256 × 224, field of view (FOV) 240 mm; 6-mm slice thickness with a 2-mm interslice gap). A bolus of gadolinium diethylenetriamine pentaacetic acid (Gd-DTPA 0.1 mmol/kg) was used.

### Immunohistochemistry

Immunohistochemical staining was performed using the EnVision™Systems (Dako, Glostrup, Denmark) according to the manufacturer’s instructions. In brief, primary antibodies (mouse anti-human LCA (M 0701), CD34 (Kit-0004), CD20 (M 0755) and CD68 (IS 609); rabbit anti-human S-100 (GA 504), CD3(Kit-0003), PAX 5(312R-1) and GFAP (Z 0334)) were incubated with brain sections followed by incubation with secondary antibodies. The sections of staining were photographed under an optical microscope (BX51, Olympus, Tokyo, Japan) and photos were captured by the software DP2-BSW(Olympus). All antibodies were purchased from Dako except that anti-CD3 and CD34 were purchased from Maixin Biotechnology Corp. Ltd (Fuzhou, China), PAX 5 was purchased from Sigma-Aldrich (Shanghai) Trading Co. Ltd.

### Evaluation of immunohistochemical staining

Immunohistochemical staining was evaluated by two experienced pathologists. Both the distribution (the percentage of positive cells) and the intensity of staining were assessed in a semi-quantitative manner. The following score system was used for recording positive cells: none (not stained) =0, focal (less than one-third of cells stained) =1, multifocal (less than two-thirds of cells stained) =2, and diffuse (most cells stained) =3. The intensity of staining was graded as follows: none (not stained) =0, mild (between 0 and 2) =1, and strong (clearly identified under scale bar 50μm) = 2. The scores for distribution and intensity were added and graded as follows: 0–2 = (-, negative), 3–5 = (+, positive).

### Statistical analysis

SPSS 22.0 software was used for statistical analysis of the data:

1. Kollomogorov-Smirnoff test was used and all measurement conformed to normal distribution. Data characteristics were described by $\bar{x}$ ± s.
2. Pearson Chi-Sqrare test was used for enumeration data: ① Four-table table, if the theoretical frequency of more than 20% cells was between 1 and 5(1≤T<5), correction for continuity of chi-square test was used. And if the theoretical frequency of more than 1 cell was less than 1 (T<1), Fisher’s test was used. ②R×C table, if T< 1 or more than 20% of the cells had T< 5, the likelihood ratio was used.
3. P<0.05 mean statistically significant.

## Results

### Clinical profiles of the patients with TDL and PCNSL

We summaried the distinguishment between TDLs and PCNSL in **Table 1**. The onset age in the TDL group was 37 ± 14 years old (**Table 2**). One of patients with TDLs was initially diagnosed as PCNSL or metastatic tumor and then treated with the gamma knife and glucocorticoid. However, a relapse occurred eight years later and demyelinating lesions complicated with radiation encephalopathy were confirmed by the brain biopsy.

**Table 1:** The distinguishment between TDLs and PCNSL.

	TDLs	PCNSL
clinical identification		
Onset age	Average age is 36	Older age
Onset time	1/4-1/3 acute onset, mostly chronic onset	insidious onset or slow progressive, rarely acute onset
Clinical features	mild symptoms in the initial stage, but more obvious than tumors, and the movement disorder is more obvious if the pyramidal tract is involved	Relatively mild, slow progressive motor involvement, even if the pyramidal tract is involved, the onset symptoms are mostly epilepsy
Onset symptoms	Mainly apathy, headache, partial limb weakness and vision loss	Mainly cognitive impairment and memory loss, but also headache and vision loss
Involved location	common white matter involvement, but cortical and subcortical white matter can also be involved; lesions can be single or multiple, most lesions are not connected, and the corpus callosum is generally not thickened	It is more likely to involve midline structures such as the thalamus, brainstem, corpus callosum, and lateral ventricle triangle, and bilateral hemisphere involvement is common
CSF	The pressure is common normal the protein is normal or increased, and MBP is positive.	The pressure is normal the protein is normal MBP is negative or slightly positive and the IL-6 and IL-10 are increased, especially the ratio.
Glucocorticoid	The lesions gradually reduced or disappeared the symptoms continued improved	Temporarily significantly reduced or disappeared (ghost cells), but new lesions may appear in other intracranial locations.
neuroimaging		
CT	Hypointense or isodense lesions and finally hypointense lesions in the increasing stage but no enhancement	Hypointense or isointense at the beginning of the onset, and develop into hyperintensity as the disease progresses, and spheroid enhancement
MRI	T1WI hypointense and T2WI hyperintense; clear lesion boundary; ring-like or C-like enhancement in acute or subacute phase, and a few clump-like enhancement; enhancement becomes less and less obvious with time flies; after 3 months goes spinal cord lesions become more obvious enhancement, TDLs are not considered; DWI in acute or subacute phase is mostly hyperintensity, and the hyperintensity gradually decreases with time flies; SWI generally has no microbleeds; the perfusion of the lesion shows hypoperfusion.	Lymphoma cells have obvious enhancement, and the enhancement becomes more pronounced over time, manifesting as “clenched fist” and “notch sign”, rarely “ring” or “semi-ring” enhancement, sometimes involving subcortical “U”-shaped fibers, along the cortical infiltrating, and “semi-ring” enhancement may appear. Sometimes necrotic cyst may appear “ring” on DWI but the enhancement is often homogeneous or mixed significant solid; in the early stage, it is mostly hypointense or isointense on DWI, and gradually become hyperintense over time; microbleeds are rarely seen on SWI; the perfusion scan shows hyperperfusion.
Pathology		
	inflammatory demyelination with relative preservation of axons. Creutzfeldt-Peters cells are key features but easily as tumor cells	Intracerebral demyelinating lesions appear at the beginning of the onset or after glucocorticoid treatment called “sentinel lesions” and then tumor cells slowly grow.

**Table 2:** Statistical between TDL and PCNSL.

	TDLs	PCNSL	P
**Number of subjects**	116	150	
**Age(years)**	37 ± 14	58 ± 13	0.000
**Gender**			0.661
**Male**	55	90	
**Famale**	61	60	
**onset**			0.094
**headache**	42 (36.2%)	35 (23.3%)	
**weakness**	18 (15.5%)	25 (16.7%)	
**numbness**	18 (15.5%)	10 (6.7%)	
**reduced vision**	11 (9.5%)	10 (6.7%)	

The onset age in the PCNSL group was 58 ± 13years, older than age in the TDLs group (p=0.000, **Table 2**). Multiple biopsies were performed in a total of 4 cases for the definite diagnosis, and 15 cases received glucocorticoid treatment before biopsy, which might change histopathological features and lead to the misdiagnosis.

There was no significantly statistical difference in onsets of TDL and PCNSL (P=0.094) (**Table 2**), suggesting that tumor was not the only diagnosis if onsets of patients with intracranial space-occupying lesions were headache, limb weakness, numbness, or reduced vision.

### Neuroimaging of TDL and PCNSL

**Figure 1:**
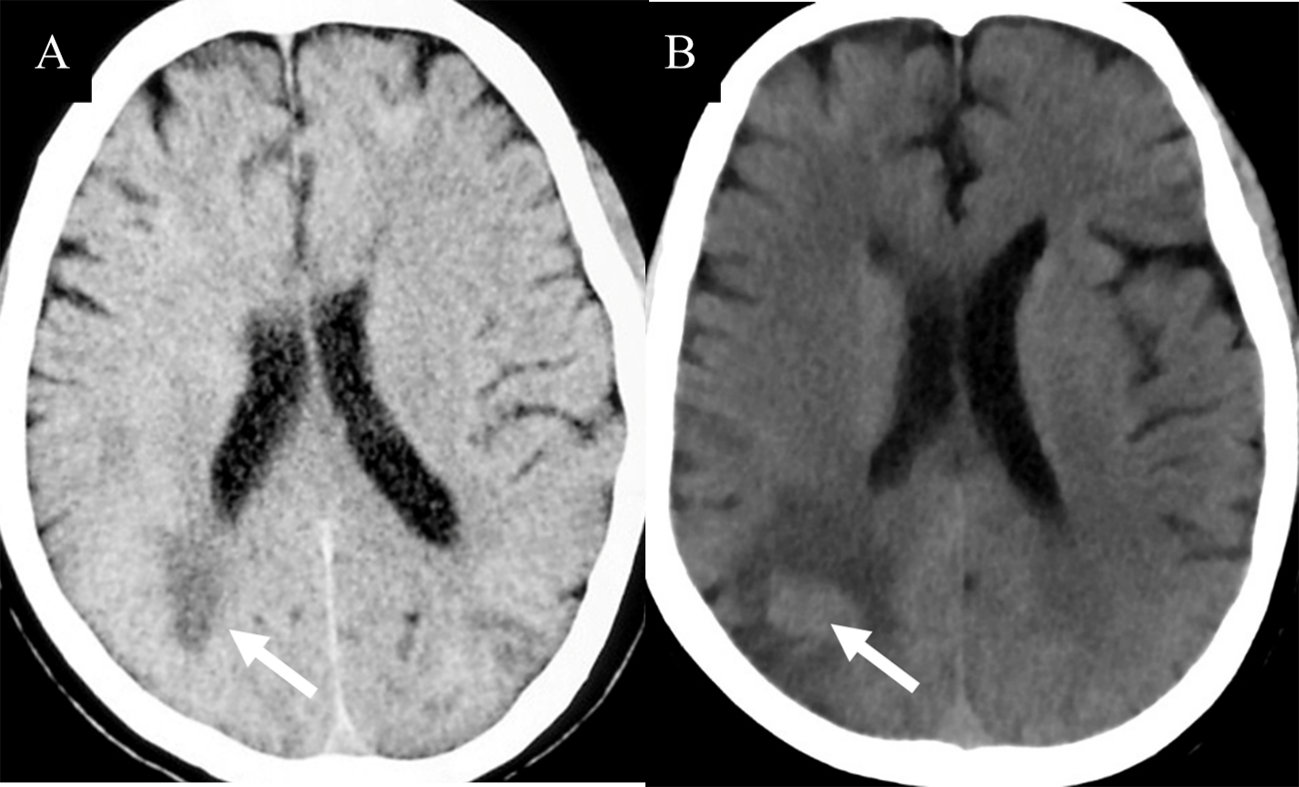
**(A)** Isodense lesion (white arrow) were detected on CT during an early disease. **(B)** One months later, brain lesions appeared hyperintense (white arrow). Pathological examinations indicated a diagnosis of PCNSL.

**Figure 2:**
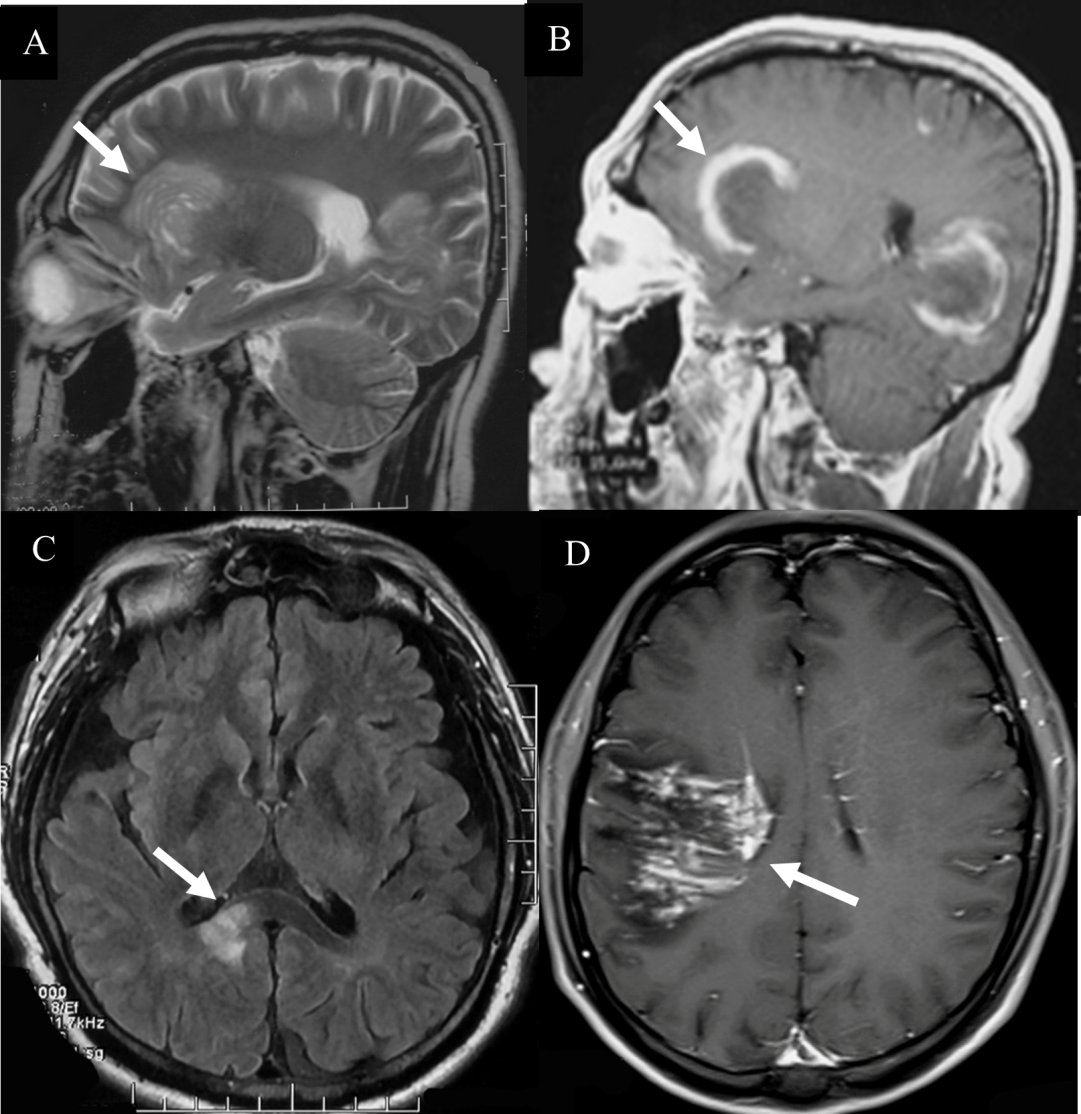
Hyperintense lesions in TDLs. **(A)** A Balo-like lesion (white arrow). **(B)** The ‘C’ like enhancement lesion (white arrow). **(C)** The lesions of TDLs without thickened corpus callosum (white arrow). **(D)** “comb sign” enhancement (white arrow).

**Figure 3:**
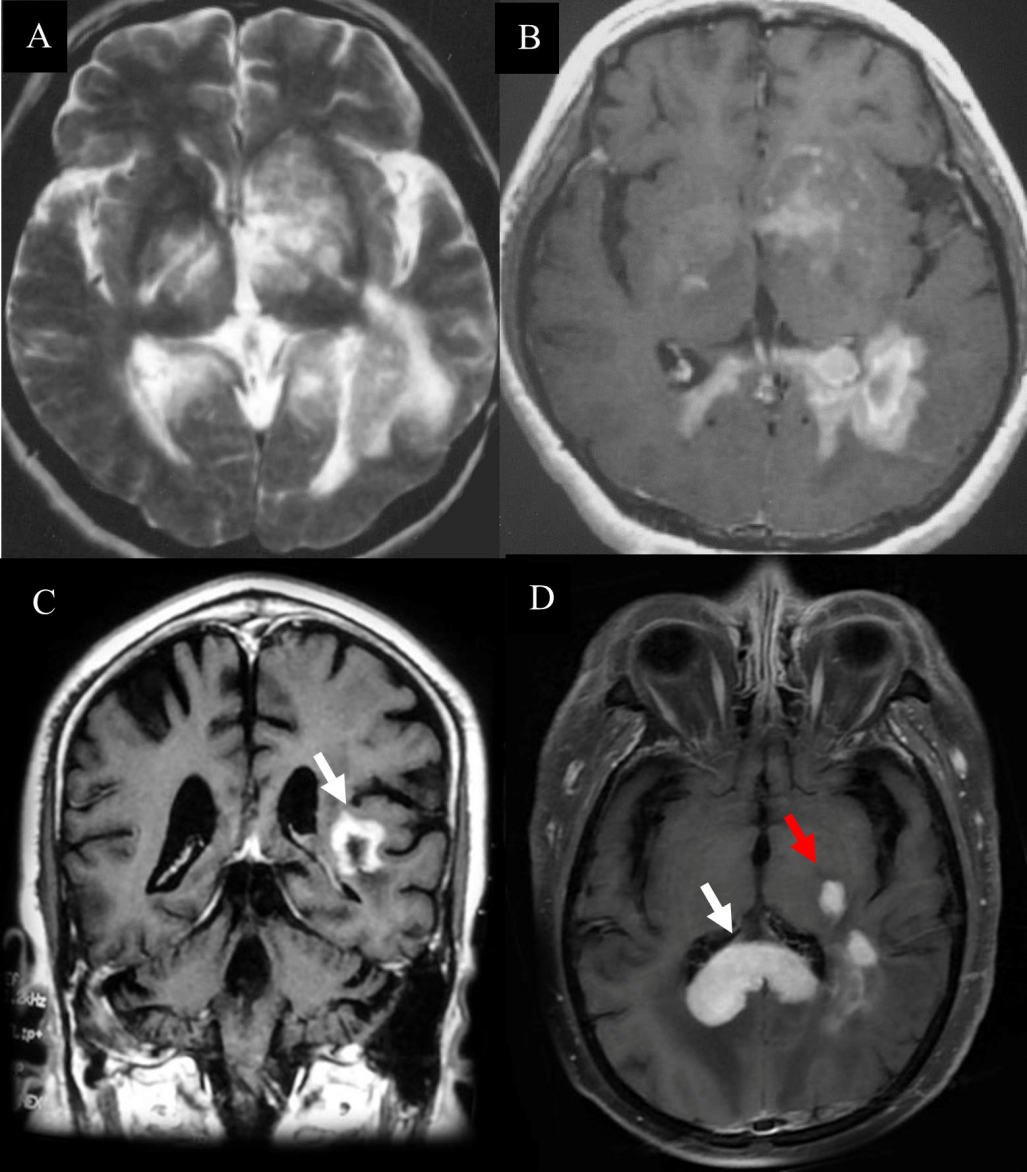
Hyperintense lesions in PCNSL. **(A)**The diffuse infiltrating lesions on T2 sequences. **(B)**The clump lesions with a ring-like enhancement. **(C)** The ‘C’ opening was not toward grey matter which differentiated from TDLs. **(D)** “kidney type”(White arrow) and “raindrop”-like (Red arrow) enhancement with thickened corpus callosum, while the lesions of TDLs without thickened corpus callosum.

The lesions of TDLs and PCNSL mainly involved in the white matter, such as lateral ventricles and centrum ovale. Upon brain CT scans, 110 cases of TDLs had hypointense lesions, and 6 cases of TDLs showed isodense lesions. Approximately 80% patients (120/150) with PCNSL had hyperdense lesions, while 12 cases had hypointense lesions with central lesion enhancement (**Figure 1**). Regarding MRI, all brain lesions of TDLs and PCNSL showed hyperintense on T_2_WI and fluid attenuated inversion recovery (FLAIR) sequences. On diffusion-weighted imaging (DWI) sequence, brain lesions in TDLs showed hyperintense, while lesions in PCNSL appeared slightly hyperintense. Brain lesions in TDLs showed flake, ‘comb sign’(**Figure 2D**), ring or ‘C’ like enhancement (**Figures 2A, B**) and the ‘C’ opening toward grey matter which differentiated from PCNSL (**Figures 2B**, **3C**). Acute lesions of TDLs (**Figure 2C**) could be easily misdiagnosed as PCNSL since its lesions showed the patchy, clump or ball-like enhancement (**Figure 3**). Furthermore, shrinking lesions following corticosteroids in PCNSL might lead to misdiagnose as TDLs, and brain lesions in TDLs with a mass effect and obvious enhancement could be easily misdiagnosed as PCNSL.

### Pathological features of TDL and PCNSL

**Figure 4:**
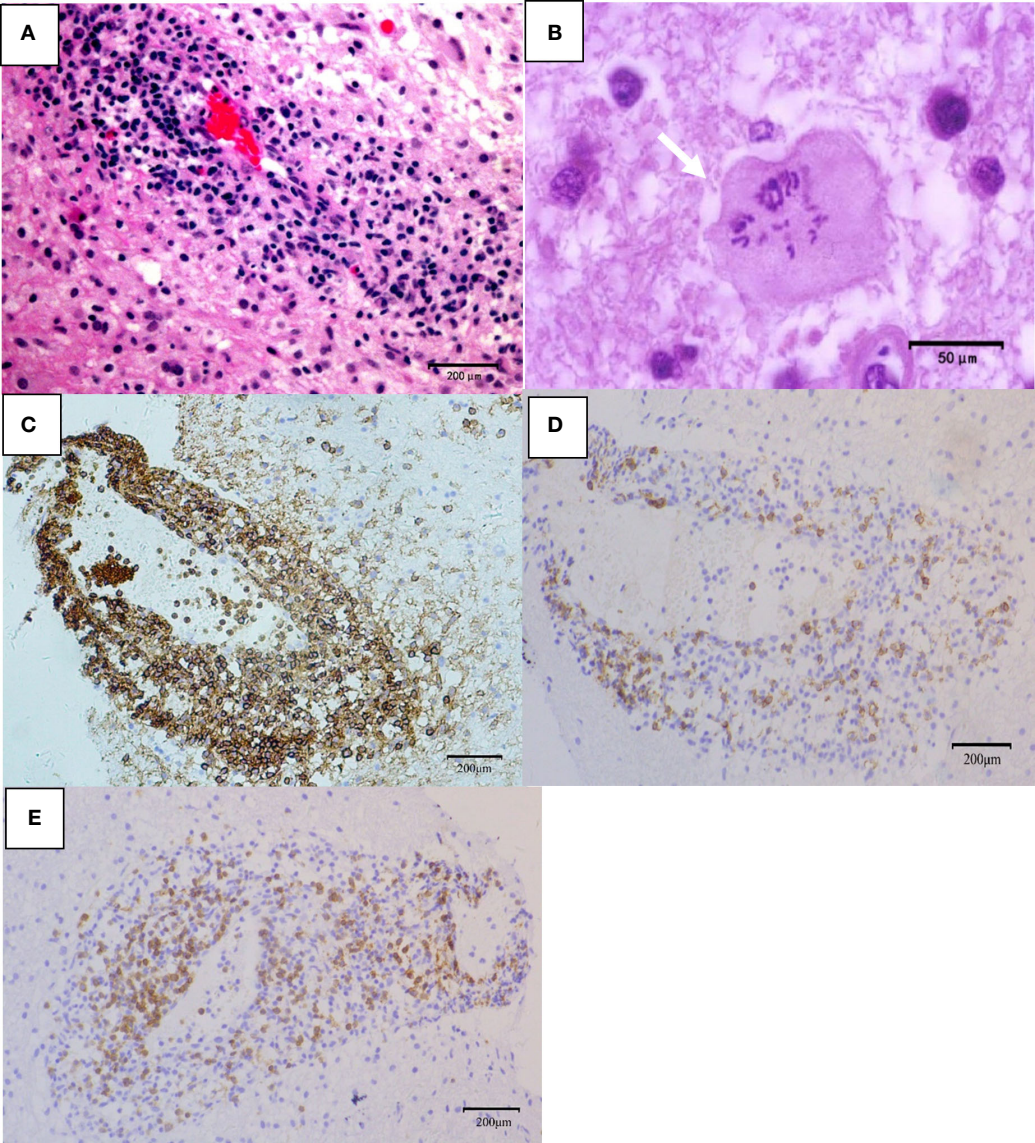
Pathological features of TDL. **(A)** Telangiectasia with hemorrhage and perivascular inflammatory cuff (hematoxylin and eosin, scale bar =200μm); **(B)** Creutzfeldt-Peters cells (white arrow, hematoxylin and eosin, scale bar =50μm);**(C)** LCA^+^ lymphocytes around the blood vessels (LCA, scale bar =200μm); **(D)** CD20^+^ B lymphocytes around the blood vessels (CD20, scale bar =200μm); **(E)** CD3^+^ T lymphocytes around the blood vessels (CD3, scale bar =200μm).

Telangiectasia with hemorrhage and perivascular infiltrates were observed in patients with TDL (**Figure 4A**). The presence of Creutzfeldt-Peters cells was another key feature in the TDL group (**Figure 4B**), with reactive astrocytes being seen in active inflammatory diseases. LCA-, CD20- and CD3-positive cells were detected in areas around the blood vessels (**Figures 4C–F**). Marked perivascular lymphocytic infiltrates were detected in TDLs. A few scattered atypical lymphocytes were observed in one case of TDLs and clinical symptoms were aggravated following corticosteroids, which made PCNSL as a possible diagnosis. However, widely depigmented myelin and positive LCA, CD20 and CD3 staining suggested a diagnosis of TDL.

**Figure 5:**
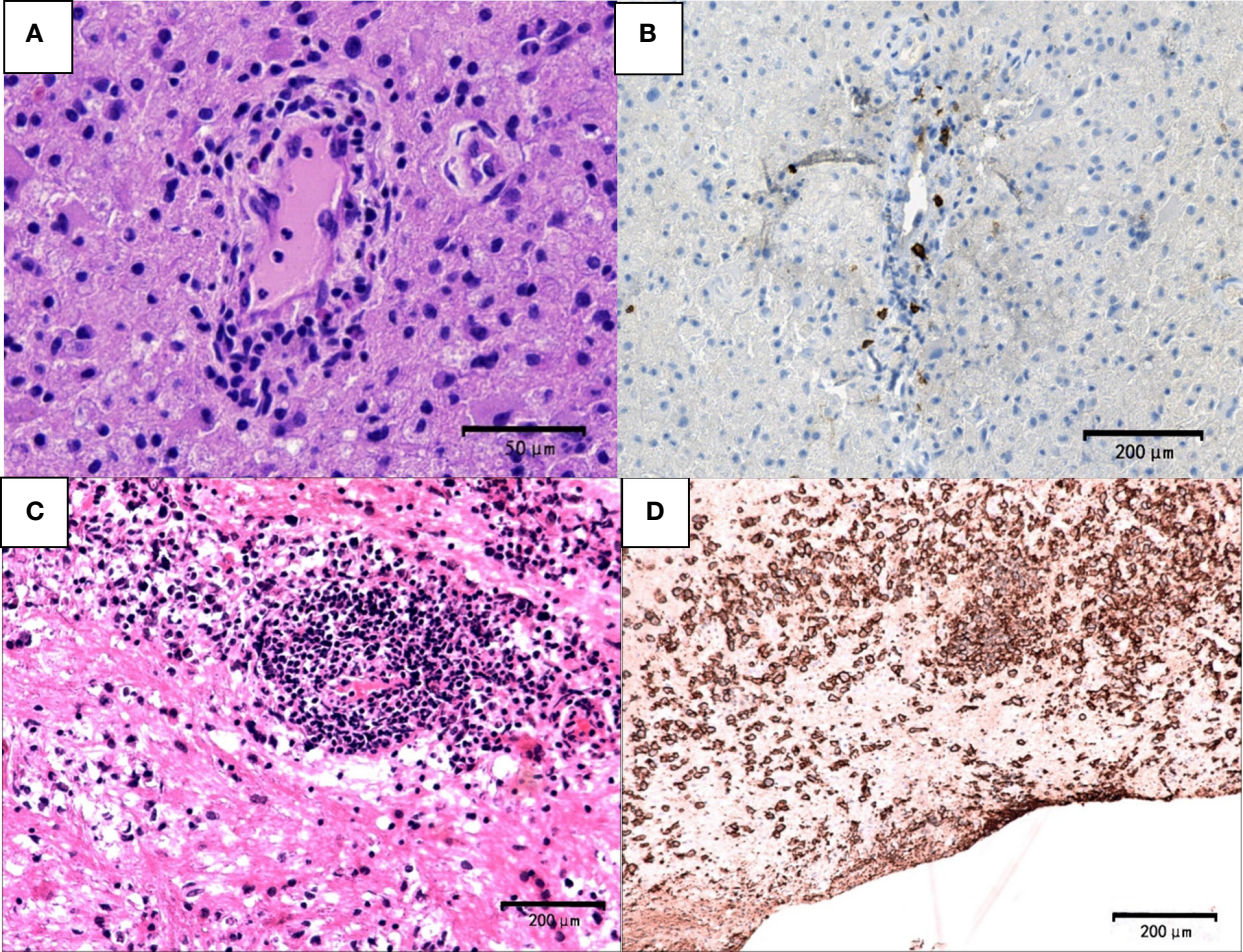
Pathological features of PCNSL. The first biopsy **(A, B)**. **(A)** demyelinated lesions as well as perivascular and parenchymal infiltration mainly composed lymphocytes (hematoxylin and eosin, scale bar =50μm). **(B)** CD20-negative cells (CD20, scale bar =200μm). The third biopsy **(C)** atypical lymphocyte infiltration around blood vessels (hematoxylin and eosin; scale bar=200μm) **(D)** CD20^+^ B lymphocytes and typical tumor cells (CD20; scale bar =200μm).

Tumor cells were absent in sentinel lesions of PCNSL which were easily misdiagnosed. Multiple biopsies were performed in some cases of PCNSL due to atypical pathological features. Hypercellular plaques of PCNSL were characterized by axonal damaged associated with perivascular lymphocytic cuffing, perilesional edema and focal degeneration. However, demyelination and tumor cells were absent. The diagnosis of TDLs was challenging. For example, a 68-year-old man in our study was received three times of biopsies. Pathological profiles of previous two biopsies showed tissue edema, focal spongiform degeneration, profound perivascular and parenchymal infiltration composed mainly of lymphocytes with the nuclear division. Immunohistochemical staining showed CD20-positive cells around the blood vessels. The patient was treated based on the diagnosis of TDL, which was not effective. The patient’s symptoms gradually deteriorated, and he thus received the third biopsy. The pathology showed that atypical lymphocytes were infiltrated around blood vessels (**Figure 5A**) and CD20-positive cells (**Figure 5B**). Furthermore, the cells were CD3-negative, indicating a diagnosis of PCNSL.

## Discussion

In our study, we disclosed neuroimaging and clinicopathological differences between TDLs and sentinel lesions of PCNSL. Onset age of patients with TDLs was younger than PCNSL. Neuroimaging features on brain CT might provide clues to make a differential diagnosis. Pathological features of PCNSL with sentinel lesions or following steroids therapy might mimic TDLs. We found that the presence of Creutzfeldt-Peters cells (could be misdiagnosed as tumor cells) may serve as an important feature in TDLs. Dynamic neuroimaging pathological and follow-up information are essential for an accurate diagnosis.

Recent studies have suggested that TDLs may be a group of relatively independent diseases, or an early manifestation of demyelinating diseases, such as multiple sclerosis (MS), neuromyelitis optica spectrum disorder (NMOSD), acute disseminated encephalomyelitis (ADEM), myelin oligodendrocyte glycoprotein antibody-associated disease (MOGAD) and clinically isolated syndrome (CIS) (13–15). Clinical manifestations mainly depend on the location and scope of the lesions. Symptoms may gradually increase or aggravate during active phase, but rarely only show epileptic seizures. Onsets of headache, slurred speech, and weakness are more common (16). In early stage, some patients may only present mental and cognitive impairment such as memory loss, unresponsiveness, apathy, which are ignored and inadvertently take MRI to find large intracranial lesions, even accompanied by peripheral edema and mass or/and enhanced lesions (17). Mass-occupying lesions are easily diagnosed as a tumor, such as PCNSL or high-grade glioma (9, 14, 18, 19). During the phase of TDLs is progressing, symptoms may gradually increase or worsen, and reduced vision may also present. When TDLs lesions are diffuse or multiple, cognitive impairment and voiding dysfunction appear, and some can occur. Although TDLs is a rare type of CNS inflammatory demyelinating disease, many different diseases need to be differentially diagnosed. Therefore, a detailed understanding of clinical imaging characteristics of TDLs will be helpful to distinguish them from tumors, avoiding unnecessary traumatic surgery and radiation therapy. Ultimately, pathological diagnosis is also the key point.

Our study included 116 patients with TDLs and 150 patients with PCNSL. All of them were pathologically confirmed in our hospital. These valuable resources might help us to further understand the characteristics of TDLs and PCNSL, especially distinguishing sentinel lesions of PCNSL from TDLs. The onset age of PCNSL was older than that of TDLs. PCNSL with isodense lesions on brain CT scans need to be differentiated from TDLs. Atypical pathological features in PCNSL might be related to the use of corticosteroids.

It was worth noting that hyperdensity in CT and boundary definition of lesions in T1 and T2 in the differences between TDLs and PCNSL. The characteristic “comb-tooth sign”, C-type or ring enhancement, double-layer enhancement, and density of DWI changes in MRI should be useful to diagnose TDLs. On the contrary, relatively uniform and significant sheet-like or spherical enhancement, “notch sign”, “pointy angle sign”, “kidney type”, and “raindrop”-like enhancement implied PCNSL.

## Conclusion

Distinguishing sentinel lesions of PCNSL and TDLs during an early disease stage remains a challenge. Due to pathological features of PCNSL are dynamic and can be associated with disease evolution, corticosteroid is not recommended for PCNSL before a definite diagnosis being made. Furthermore, repeated biopsies may be needed in some patients with PCNSL and TDLs for a definitive diagnosis.

## Limitation

Although the large sample was analyzed in our research, a single-center design should be stated as a limitation. Limited clinical profiles, imaging profiles, laboratory, and pathological findings in patients with TDL and PCNSL prevented further analysis. In future, we will continue to collect patients from multiple centers for further analysis.

## Data availability statement

The original contributions presented in the study are included in the article/supplementary material. Further inquiries can be directed to the corresponding authors.

## Author contributions

CS, JH and YL acquired the clinical data, reviewed the literature, and drafted the article. FQ and JL designed the study, supervised the initial drafting, and critically revised the article. XQ and CL collected and analyzed the clinical data. All authors contributed to the article and approved the submitted version.

## Conflict of interest

The authors declare that the research was conducted in the absence of any commercial or financial relationships that could be construed as a potential conflict of interest.

## Publisher’s note

All claims expressed in this article are solely those of the authors and do not necessarily represent those of their affiliated organizations, or those of the publisher, the editors and the reviewers. Any product that may be evaluated in this article, or claim that may be made by its manufacturer, is not guaranteed or endorsed by the publisher.
